# Suppressing the Encoding of New Information in Memory: A Behavioral Study Derived from Principles of Hippocampal Function

**DOI:** 10.1371/journal.pone.0050814

**Published:** 2013-01-16

**Authors:** Sinéad L. Mullally, Shane M. O'Mara

**Affiliations:** Trinity College Institute of Neuroscience and School of Psychology, Trinity College Dublin, Dublin, Ireland; University of British Columbia, Canada

## Abstract

Cognitive processes do not occur in isolation. Interactions between cognitive processes can be observed as a cost in performance following a switch between tasks, a cost that is greatest when the cognitive requirements of the sequential tasks compete. Interestingly, the long-term mnemonic goals associated with specific cognitive tasks can also directly compete. For example, encoding the sequential order in which stimuli are presented in the commonly-utilised 2-Back working memory (WM) tasks is counter-productive to task performance, as this task requires the continual updating of the contents of one's current mental set. Performance of this task consistently results in reduced activity within the medial temporal lobe (MTL), and this response is believed to reflect the inhibitory mnemonic component of the task. Conversely, there are numerous cognitive paradigms in which participants are explicitly instructed to encode incoming information and performance of these tasks reliably increases MTL activity. Here, we explore the behavioural cost of sequentially performing two tasks with conflicting long-term mnemonic goals and contrasting neural profiles within the MTL. We hypothesised that performing the 2-Back WM prior to a hippocampal-dependent memory task would impair performance on the latter task. We found that participants who performed the 2-Back WM task, prior to the encoding of novel verbal/face-name stimuli, recollected significantly fewer of these stimuli, compared to those who had performed a 0-Back control task. Memory processes believed to be independent of the MTL were unaffected. Our results suggest that the inhibition of MTL-dependent mnemonic function persists beyond the cessation of the 2-Back WM task and can alter performance on entirely separate and subsequently performed memory tasks. Furthermore, they indicate that performance of such tasks may induce a temporarily-sustained, virtual lesion of the hippocampus, which could be used as a probe to explore cognitive processes in the absence of hippocampal involvement.

## Introduction

The critical role of the human hippocampus in supporting memory has long been recognised [1], [2] and this role continues to be elucidated by neuropsychological [3], [4] and neuroimaging approaches [5], [6], [7]. Significantly, studies utilising these latter techniques have consistently linked increases in markers of neuronal activity [i.e. regional cerebral blood flow (rCBF: PET) and blood oxygenation level dependence (BOLD: fMRI)] within the medial temporal lobes to the successful encoding or recollection of to-be-remembered information [8], [9], [10], [11], [12]. More recently however, the human hippocampus has also been implicated in memory inhibition [13]. Specifically, it has been repeatedly demonstrated that it is possible to actively suppress previously learned material, resulting in the apparent loss of this information from long-term memory [14], an ability that emerges in late childhood [15] and declines in the aging brain [16]. Moreover, successful inhibition of the memory trace is associated with an increase in the BOLD signal response in the dorsolateral prefrontal cortex (DLPFC) and a corresponding decrease (or negative BOLD response) within the hippocampus [13]; a decrease which appears to be under the control of the prefrontal cortex [17]. Therefore, just as a positive BOLD response in the human hippocampus is often considered to represent increased mnemonic processing, a decrease or negative BOLD response may be associated with the active and specific inhibition of memory [13], [17], [18].

Interestingly, this neural profile is not unique to tasks in which the active suppression of the memory trace is explicit. For instance, versions of the *n*-Back working memory task which present participants with sequences of highly-familiar stimuli (such as numbers or letters) are believed to contain a similar, yet implicit, inhibitory component. In such tasks participants are required to continually update the contents of their working memory, while simultaneously responding to stimuli presented *n* trials previously [19], [20], [21]. However, whilst this updating entails the assimilation of new information into working memory, it also requires the removal of the older, now task-irrelevant, information from the current mental set. If this new information is sufficiently similar to the older, out-going information, an optimal task strategy is to actively inhibit the memory trace of the older information before it is encoded into long-term memory [20]. This strategy minimises any potential interference with new incoming information, and leads ultimately to the successful completion of the task at hand. Unsurprisingly, the successful performance of such versions of the *n*-Back WM task is also associated with a positive BOLD response in DLPFC and a corresponding decrease in rCBF [20], [22], [23] or a negative BOLD response [19], [21], [24], [25], [26] within the medial temporal lobes. Thus, tasks requiring inhibition of a memory trace from long-term memory at both an automatic (i.e. the *n*-Back WM task) or an intentional level (i.e. retrieval induced forgetting paradigms), are associated with decreased activity within the medial temporal lobes. This neural response stands in marked contrast to that associated with active mnemonic processes [8], [10], [11], [12], [27], [28].

Identifying tasks with such distinct hippocampal activity profiles and contrasting long-term mnemonic goals therefore enables one to pose an interesting question: i.e., does performing two such tasks in sequential order impair performance on the latter of the two tasks? Task-switch paradigms routinely report a behavioural cost of switching between successive tasks, especially when the cognitive requirements of the sequential tasks compete [29]. This is believed to be, in part, due to a failure to disengage the cognitive processes and neural mechanisms supporting the initial task, which in turn, are inappropriate or suboptimal to support performance on the second task (see also [30]). The specific inhibition of mnemonic processes via performance of an *n*-Back or active memory suppression task could thus potentially impinge on the subsequent performance of hippocampal-dependent memory task, given that the mnemonic goals of such tasks appear to oppose one another. We hypothesised that prior performance of a ‘mnemonic-inhibitory’ task (in this case, the *n*-Back WM Task in which the MTL appears to be in a temporarily-inhibited state), followed by the immediate performance of a more traditional MTL-dependent memory task, would induce a systematic cost (or deficit) on the latter task. This cost or deficit is most likely attributable to the inappropriate and conflicting carry-over of the initial ‘neural task-set’ engaged by successful performance of the *n*-Back task. To test this hypothesis, we conducted a series of experiments which quantified the behavioural impact of antecedent 2-Back WM task performance on MTL-related memory processes using MTL-dependent (i.e. verbal and associative learning) paradigms. We predicted that prior 2-Back WM task performance would impose a behavioural cost on the subsequent acquisition of information depending on MTL activation (Experiments 1 & 2), and we hypothesised that MTL-independent memory processes (i.e. item-priming) would be unaffected by this manipulation (Experiment 1). We also hypothesised that a similar behavioural cost should be evident in the opposite direction, i.e., that prior performance of a heavily-dependent MTL task should impair subsequent performance of the 2-Back WM task (Experiment 3).

## Materials and Methods

### Ethics Statement

Ethical approval was granted by the School of Psychology Ethics Committee, Trinity College Dublin, Ireland. Written informed consent was obtained from all participants prior to the commencement of the study and all testing was performed in accordance with local ethics guidelines.

### Participants

#### Experiment 1

One hundred healthy Trinity College Dublin undergraduate and postgraduate students (with normal or corrected-to-normal vision) participated in Experiment 1. Half were assigned to the 0-Back control condition (n = 50, males = 10), and half were assigned to the 2-Back experimental condition (Experiment 1: n = 50, males = 10) condition. No between-group differences were evident with respect to age [0-Back = 21.8 years (±4.5 years); 2-Back = 21.2 years (±2.9 years), p = 0.48].

#### Experiment 2

Using the same recruitment criteria, 24 participants were assigned to either the 0-Back control condition (n = 13, males = 7) or the 2-Back experimental condition (n = 11, males = 5). These two groups were equated with respect to age [0-Back = 20.8 years (±2.4 years); 2-Back = 21.8 years (±3.3 years), p = 0.38], time spent in full-time education [0-Back = 15.8 years (±2.1 years); 2-Back = 16.9 years (±3.3 years), p = 0.32], predicted full-scale IQ (measured using the National Adult Reading Task [31]) [0-Back = 123.2 (±2.1), 2-Back = 123.8 (±1.1), p = 0.52], and performance on the Digit Span Test [0-Back = 14.2 (±2.1), 2-Back = 14.0 (±1.95), p = 0.85].

#### Experiment 3

Again, using identical recruitment criteria, 56 participants were assigned to either the control condition (n = 29, males = 15) or the experimental (n = 27, males = 13) condition. No between-group differences were observed in terms of participant age [control group = 20.22 years (±1.4 years); experimental group = 21.10 years (±2.3 years); p = 0.16].

In all three experiments, participants received monetary compensation or course credit (undergraduate psychology students) in return for their participation.

### Tasks and Experimental Design

In both Experiment 1 and Experiment 2, participants in the control condition performed the 0-Back task, whilst those in the experimental condition performed the 2-Back WM task. The dependent variables were measured by performance on the verbal recall and recognition tasks (Experiment 1) and on the face-name learning and recall task (Experiment 2). In Experiment 3, all participants performed the 2-Back WM task as in this paradigm 2-Back WM task performance was the dependent variable of interest.

#### The *n*-Back Task

**Figure 1:**
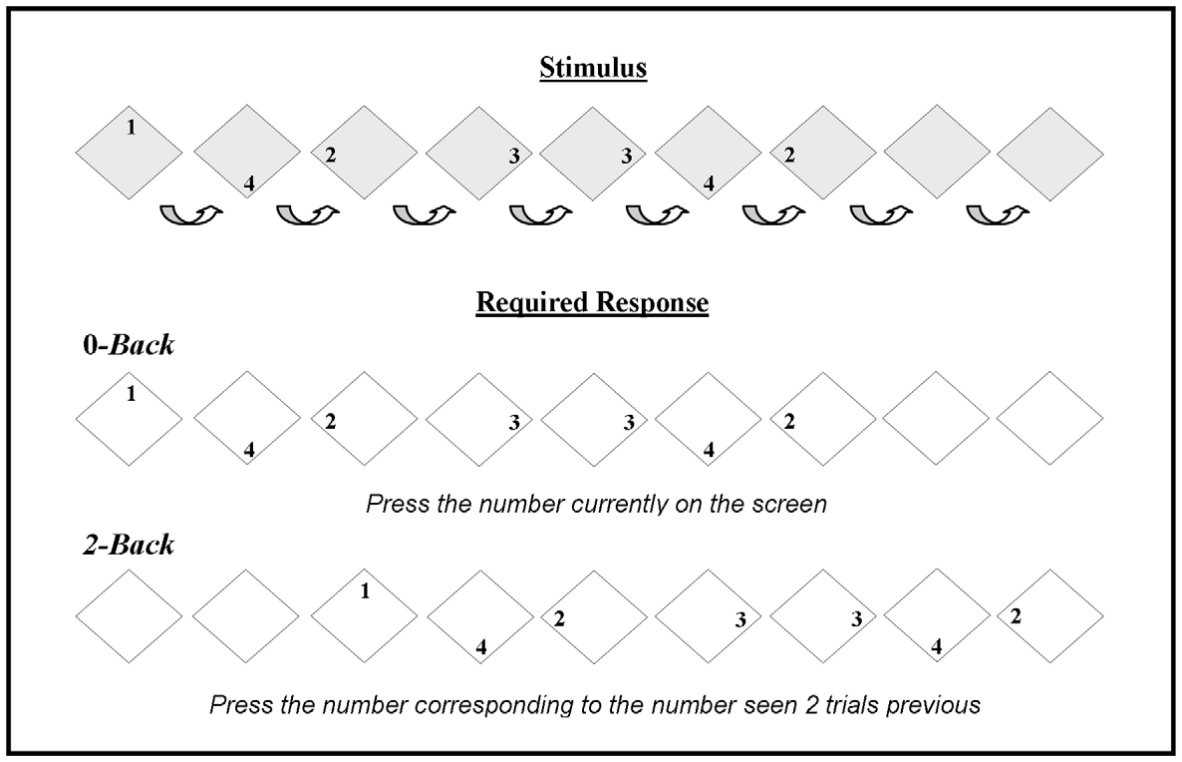
The *n*-Back task paradigm. In both conditions stimulus presentation was identical i.e. stimuli remained on-screen for 1800 ms, followed by a blank screen (ISI = 200 ms). Stimuli were presented in a pseudo-random sequence i.e. every nine randomly presented numerical stimuli were followed by two blank diamond arrays. This signaled a pause in the number sequence. Each *n*-Back (0-Back or 2-Back) block consisted of 77 visual stimuli presentations (11 visual stimuli x 7 number sequences) and total block duration was 154 seconds. *0-Back Control Task:* Participants were required to respond by pressing the number corresponding to the number in the diamond on screen. *2-Back WM Task*: Participants were required to respond by pressing the button corresponding to the number presented 2 diamonds previously. (Blank screen omitted between stimuli).

The design of both *n*-Back tasks (i.e. the 0-Back and 2-Back tasks) was identical to that previously reported in the literature [21], whereby participants viewed a consecutive stream of diamond shaped stimuli (duration = 1800 ms/stimulus; ISI = 200 ms) containing the digits “1”, “2”, “3”, “4” or no numerical information (see Figure 1). All responses were made using a Cedrus RB-530 response pad (Cedrus, San Pedro, CA), where the spatial layout corresponded to the spatial arrangement of the stimuli. Participants performing the 0-Back sensori-motor control task simply pressed the number corresponding to the number currently on screen, whilst those performing the 2-Back task pressed the number that was presented two trials previously. The 2-Back task therefore required the maintenance of the current number and the last two numbers in working memory, and the continuous updating of this running count with the presentation of each successive stimulus. Each *n*-Back block lasted approximately 154 seconds. Accuracy and reaction times were recorded.

#### Experiment 1

**Figure 2:**
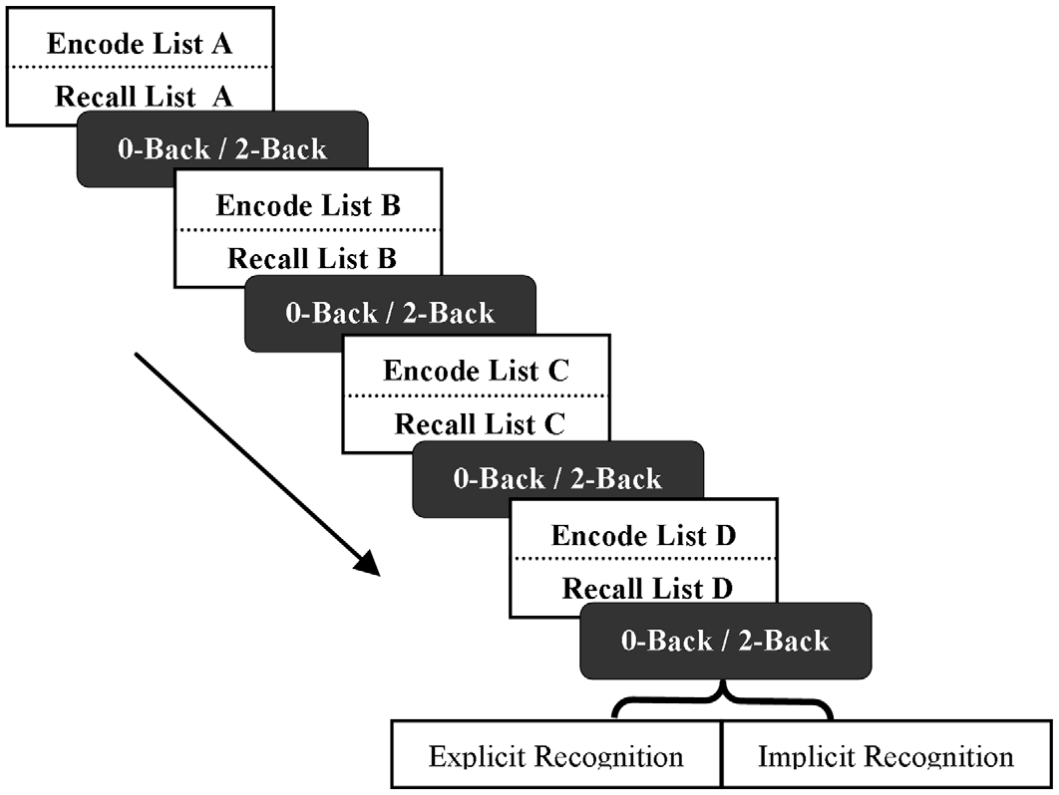
Task design: Experiment 1. Blocks of the 0-Back control task or the 2-Back WM task (154 seconds) were embedded between the four verbal learning (∼120 seconds) and recall (∼180 seconds) trials and prior to the hippocampal-dependent/hippocampal-independent verbal recognition task (∼180 seconds). Half of the participants performed the hippocampal-dependent/direct recognition task, whilst the other half performed the hippocampal-independent/indirect recognition task.

Here we aimed to assess the behavioural impact of antecedent 2-Back WM task performance on subsequent verbal learning and recollection. Half of the participants were assigned to the 2-Back group, whilst the other half were assigned to the 0-Back control group. Blocks of the *n*-back task were interwoven with four blocks of the verbal learning, and a verbal (hippocampal-dependent or hippocampal-independent) recollection task (see Figure 2).

The verbal learning task consisted of four word lists (A, B, C, and D) containing 15 words per list. These words were two-syllable words, selected from the Toronto Word Pool [32], and scaled for imagery (List A = 5.6 (±0.6); List B = 5.6 (±0.7); List C = 5.7 (±0.6); List D = 5.6 (±0.7); p = 0.91), concreteness (List A = 5.7 (±0.7); List B = 5.8 (±0.8); List C = 5.7 (±0.5); List D = 5.8 (±0.5); p = 0.88), frequency (List A = 101.7 (±53.5); List B = 81.3 (±32.1); List C = 81.3 (±32.1); List D = 130.5 (±63.1); p = 0.06), and number of letters (List A = 6.5 (±1.1); List B = 6.5 (±1.1); List C = 5.9 (±0.92); List D = 6.5 (±0.92) ; p = 0.22). A pilot study ensured that the difficulty levels of these lists were equated. Each list was presented item-by-item (black TNR 48 point font; duration = 1000 ms/word; ISI = 1000 ms) and was followed by a test of free recall (see Figure S1: Panel A). In the subsequent hippocampal-dependent and hippocampal-independent recognition tasks, the 60 previously studied words (List A+List B+List C+List D) were randomly dispersed among the 60 unseen “distractor” items. Each item was then presented individually on screen (see Figure S1: Panel B). In the direct, hippocampal-dependent recognition task, participants were required to identify target items (i.e. the previously studied words) and to reject “distractor” items. Stimuli remained on screen until participants verbalised their decision, after which they made a button press (to progress to the next trial). Participants were encouraged to respond as quickly and accurately as possible. In the indirect, hippocampal-independent recognition task, no direct link was made between the previously presented word lists. Instead participants were presented with the 120 verbal stimuli described above in fragmented form (see Figure S1: Panel C). Such item-priming tasks are believed to be equated, in terms of task reliability, with the corresponding hippocampal-dependent recognition paradigm described above [33]. The fragmentation was achieved by superimposing a partially-transparent (50%), white, rectangular mask over each word. Each stimuli was presented sequentially on screen (duration = 1000 ms/stimulus; ISI = 500 ms). Participants were instructed to read each word. Words which participants successfully read aloud were considered to be correctly identified.

The overall experimental design was as follows: to begin, and prior to performing any *n*-back task blocks, all participants encoded and subsequently recalled word list A. List A thus served as a baseline control measure of verbal learning. Next, participants were trained on the relevant *n*-Back task version (0-Back or 2-Back depending on condition). A single block of this *n*-Back task was then performed immediately prior to the encoding and immediate recall of lists B, C, and D (see Figure 2). Significantly, the switch between each discrete task block (i.e. between performance of the *n*-Back task blocks, and the verbal learning and recall task) was self-paced, with both the verbal list learning and *n*-Back task blocks initiated by participants using a button press. We hypothesised that participants, who performed the 2-Back WM task prior to the presentation of lists B, C, and D, would recall fewer words from each of these lists relative to those who performed the 0-Back control task. Once this phase was completed, all participants performed a final block of the *n*-Back task (0-Back or 2-Back version, as appropriate) followed by the verbal recognition task. Here, participants were divided into two further groups, with half of the participants in each condition performing the direct, hippocampal-dependent, recognition paradigm, whilst the other half performed an identification task for words that are presented in visual noise. Typically, words that have been previously viewed tend to be identified more often than words that had not been previously viewed; such findings are taken as an indirect indication of recognition, and do not require the hippocampus. Therefore, we can contrast the influence of the *n*-Back task on hippocampal-dependent and hippocampal-independent recognition paradigms (n = 25 per group; see Figure 2). Again, we predicted that participants in the 2-Back condition would recognise fewer target words than those in the 0-Back condition when recognition was tested on the hippocampal-dependent task, but that no between-group impairment would be evident when the hippocampal-independent assessment was used [34], [35], [36], [37], [38].

#### Experiment 2

This next experiment aimed to explore the impact of prior 2-Back, relative to 0-Back, task performance, on interwoven blocks of associative learning (again using a between-group design). Such tasks require participants to form an association between an unfamiliar face stimulus and a second arbitrary piece of information (e.g. a name). This process has been found to require the involvement of the hippocampal formation [39], [40], [41], [42] in an ecological-valid context, and the task utilised here was based on a previous fMRI face-name learning paradigm which observed strong hippocampal activity during both the learning and recall of the face-name pairs [43]. In total, participants viewed eight female faces (selected from a college yearbook and presented in black and white with all hair removed), presented serially with a corresponding name (duration = 3500 ms/stimulus; ISI = 500 ms; see Figure S2: Panel A). All names were two syllables, English female names, selected from records of the most popular baby names in Ireland and Britain in the 1970s and 1980s. Participants were instructed to memorize the name corresponding to each face. After participants viewed all eights face-name pairs, they then performed a visual attention task prior to recollection. The aim of this task was to provide a short distraction between the encoding and retrieval of the face-name pairs. Here, participants viewed a centrally-located fixation cross, enclosed within the black outline of a circle, and had to respond (via a button press) every time this fixation cross changed into a solid black circle (see Figure S2: Panel B). This occurred randomly every 1–5 seconds (duration = 300 ms). Each visual attention task block lasted a total of 154 seconds. Finally, face-name recollection was assessed by presenting participants with the eight faces presented in random order, without accompanying names (see Figure S2: Panel C). Again, each face was presented for 3.5 seconds (ISI = 500 ms), during which time participants were required to vocally recall the names corresponding to each of the eight faces.

**Figure 3:**
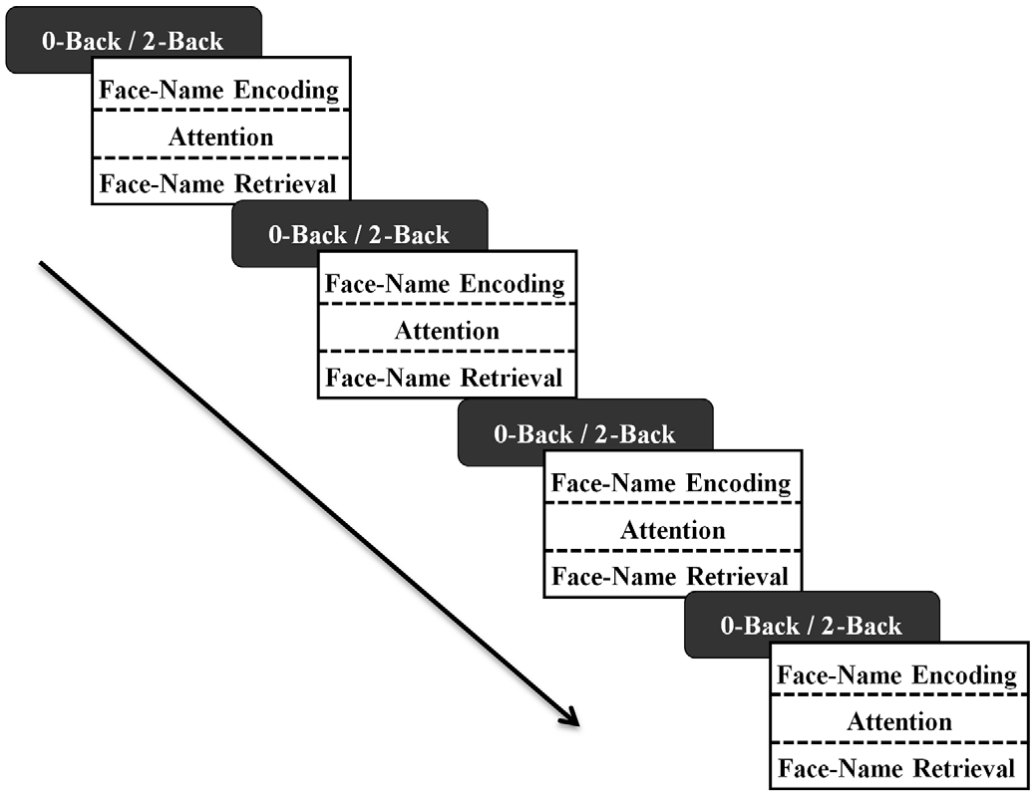
Task design: Experiment 2. Participants performed the 0-Back control task or the 2-Back WM task (duration = 154 seconds) depending on their assigned experimental condition. Following performance of the relevant *n*-Back task block, all participants performed a face-name encoding task (duration = 32 seconds). This was followed by a 154 second visual attention task block, and finally, a block of face-name pair retrieval (duration = 32 s). This sequence was repeated a total of 4 times, with participants viewing the same face-name combinations in each repetition.

As in experiment 1, the encoding blocks were preceded by blocks of the *n*-Back task (0-Back or 2-Back version depending on condition; 154 s/block) and the switch between the *n*-Back and face-name learning trials was self-paced. As illustrated in Figure 3, the task sequence was as follows: *n*-Back task, face-name encoding, visual attention task, face-name recall. This sequence was repeated a total of four times, with the same eight face-name pairs studied successively across each block. Again, we hypothesised that participants who performed the 2-Back WM task prior to the face-name task would recall significantly fewer face-name pairs than participants who performed the 0-Back control task.

#### Experiment 3

In this final experiment, we explored whether prior performance of a MTL-dependent task (e.g. the face-name learning task) negatively impacted on subsequent performance of the 2-Back WM task. Therefore, in the experimental condition, blocks of the face-name learning task (see Experiment 2 and Figure S2) were interwoven with blocks of the 2-Back WM task. Participants in the control condition performed a task of sustained attention (the Sustained Attention to Response SART Task [44]) in place of the face-name learning and recall task. In this task, participants viewed a random series of digits; ‘1’ through to ‘9’. Each digit remained on screen for a duration of 250 ms (ISI = 900 ms) during which time participants were required to make a button-press response for each presented digit (‘go trial’), except when presented with the digit ‘3’ (‘no-go trial’; see Figure S3). This task was selected as it is not believed to engage MTL regions (in the same way as the face-name or verbal memory tasks do) or inhibit long-term mnemonic processes (as is the case in the 2-Back task).

**Figure 4:**
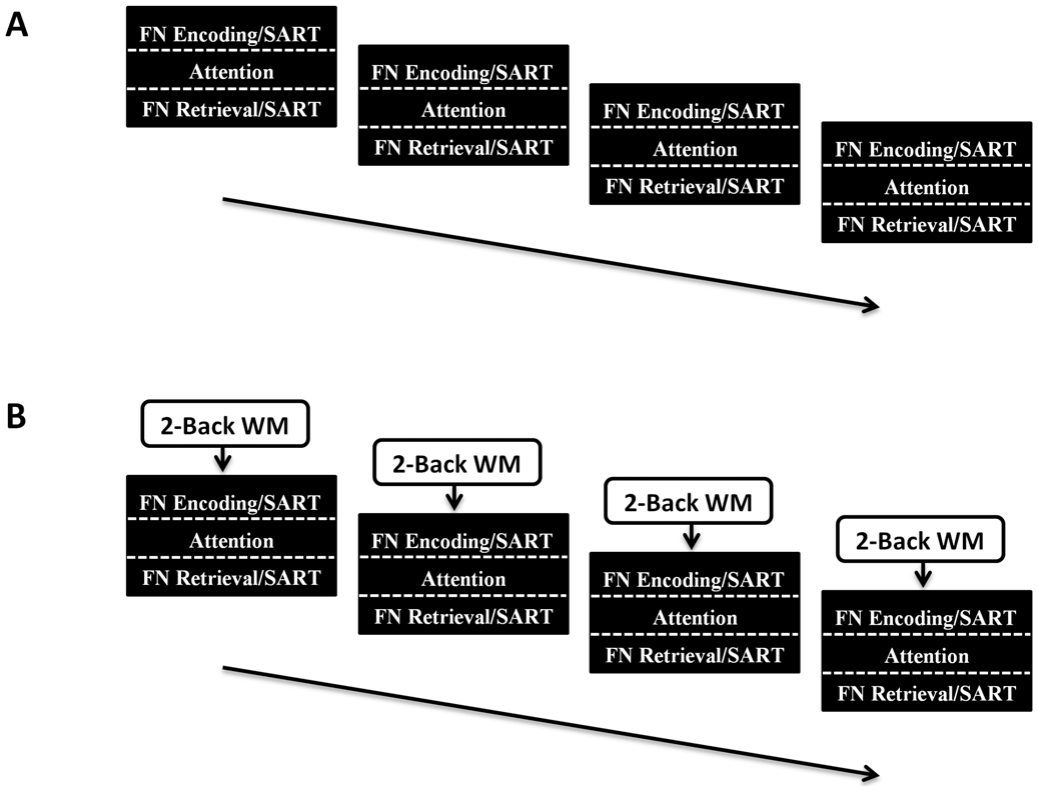
Task Design: Experiment 3. *Panel A: Phase 1*. The experimental group performed four blocks of the face-name task, which consisted of an encoding phase (10 face-name pairs; duration = 40 s) and a subsequent retrieval phase (duration = 40 s), separated in time with a ‘distractor’ task of visual attention (duration = 154 s). The control group performed the Sustained Attention to Response Task (SART), which was again divided into two blocks of 40 seconds (corresponding to the face-name encoding and face-name retrieval task blocks in the experimental condition), which were also separated with the ‘distractor’ task of visual attention (duration = 154 s). This sequence was repeated a total of four times. *Panel B: Phase 2*. This phase was identical to Phase 1, with two exceptions. Firstly, the experimental group was presented with novel face-name pairs to encode and retrieve. Secondly, prior to each of the four task blocks, all participants (in both the experimental and control groups) performance the 2-Back WM task (duration = 154 s/block). Performance on this task was the dependent variable of interest.

As in the previous experiments, both groups of participants performed a fixed sequence of interwoven tasks blocks. Here, however, the two groups of participants also performed an initial phase (i.e. Phase 1) that did not contain the task of interest (i.e. the 2-Back WM task; see Figure 4: Panel A). The aim of this phase was to up-regulate the neural-task set pertaining to either the Face-Name task [43] or the Sustained Attention to Response task [45]. The structure of this task phase was as follows: a block of either the Face-Name encoding task or another block of the SART (duration = approximately 40 seconds), was followed by the Visual Attention Task (described in Experiment 2; duration = 154 seconds), which was in turn followed by either the recall component of the Face-Name task or the SART (duration = approximately 40 seconds). This sequence was repeated a total of four times, with the same ten face-name pairs studied across the successive task blocks in the experimental condition. Once this phase was completed, participants immediately commenced Phase 2, which was identical to Phase 1 with the exception, that here all participants also performed the 2-Back WM task (see Figure 4: Panel B). The sequence of tasks in phase 2 was as follows: performance of the 2-Back WM task (block duration = 154 seconds) was followed by a block of either the encoding phase of the face-name task (which contained ten novel face-name pairs) or the SART (duration = approximately 40 seconds). Next, all participants performed the Visual Attention Task (duration = 154 seconds), and finally, either the recall phase of the face-name task, or another block of the SART (duration = approximately 40 seconds). Again, this sequence was repeated a total of four times and the same ten face-name pairs were studied across the four successive task blocks.

### Data Analysis

Statistical analyses were carried out using SPSS (version 14) for PC. Alpha was set at *P*<0.05 and all data are expressed as means ± standard error (± S.E.), unless specified otherwise. Analysis of Variance (ANOVA) was the primary statistical tool. Mixed-factorial ANOVAs were used to compare performance across the repeated blocks and between the two groups. Therefore, changes across the within-subject levels (i.e. Block), across the between-subject level (i.e. Condition) and the combined effect of both factors could be determined (i.e. Block X Condition interaction). When a significant main effect (i.e. an overall between-subject effect) was observed, further one-way ANOVAs were conducted. This allowed comparisons between-groups at each of the individual within-subject levels to be made.

## Results

### Experiment 1

#### Free Recall

**Figure 5:**
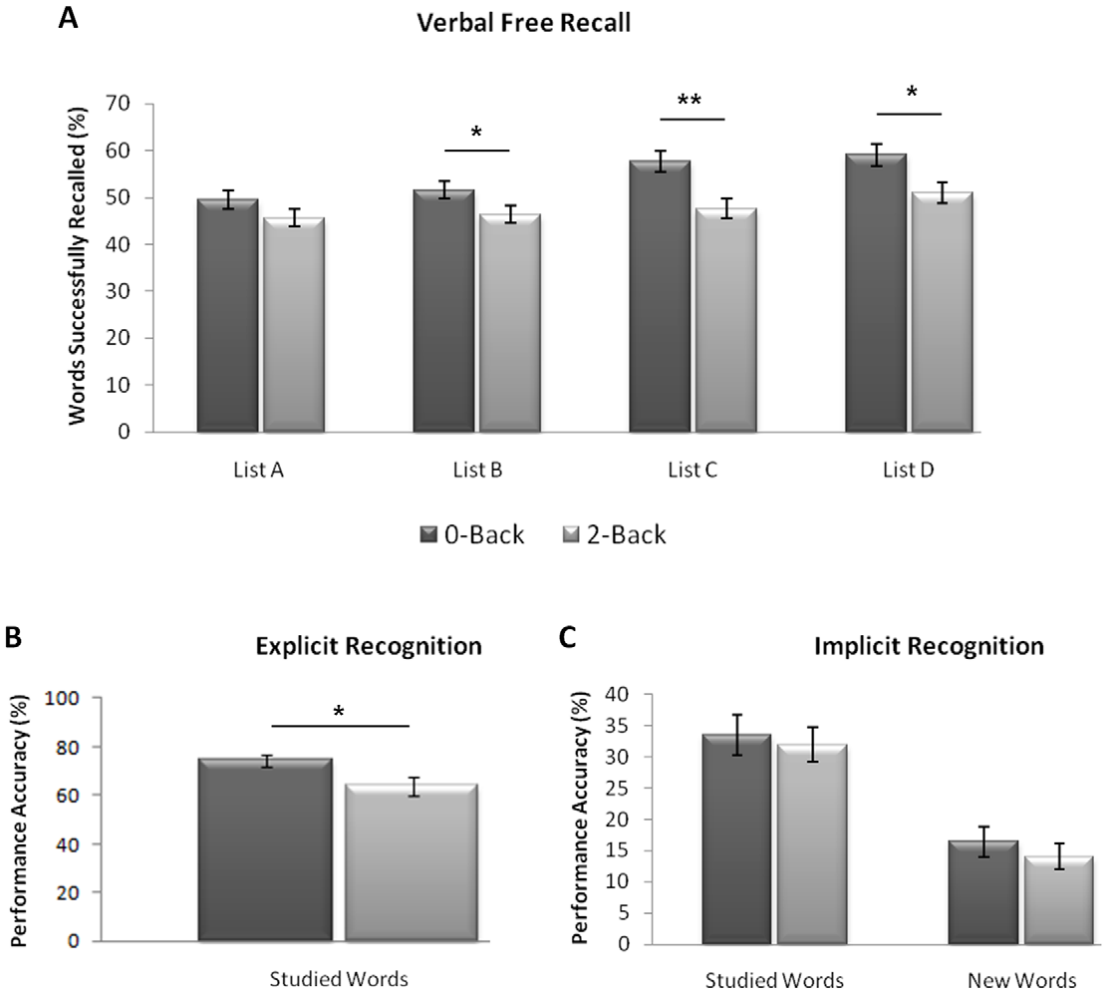
Results: Experiment 1. *Panel A*: *Free Recall*. Participants who performed the 2-Back WM task recalled significantly fewer words from Lists B, C and D. *Panel B*: *Hippocampal-Dependent/Direct Recognition* (the proportion of previously studied words successfully recognized). Individuals in the 2-Back condition performed significantly less accurately than those in the 0-Back condition. *Panel C*: *Hippocampal-independent/Indirect Recognition*. The proportion of studied and unstudied words successfully read aloud by participants. All participants successfully read more of the studied than the unstudied words. However, no performance difference was observed between the two conditions. Error bars indicate SEM. * *P*≤0.05; ** *P*<0.01.

Overall, participants who performed the 2-Back WM task prior to the encoding of Lists B, C and D recalled significantly fewer words than those who performed the 0-Back control task [F(1,98) = 8.4; p<0.01]. No Group X Word List interaction was observed [F(3, 294) = 1.83; p = 0.14]. Multiple one-way ANOVAs indicated that those allocated to the 2-Back condition recalled significantly fewer words from List B [F(1,98) = 3.08; p = 0.05], List C [F(1,98) = 10.53; p<0.01], and List D [F(1,98) = 6.21; p<0.05]. No between-group difference was observed at List A (Baseline) [F (1, 98) = 2.02; p = 0.16] (see Figure 5: Panel A).

Interestingly, this performance difference between the two groups appeared to reflect a progressive increase in the number of words successfully encoded and recalled across the four word lists (A to D) by the 0-Back control group [F(3,147) = 10.92; p<0.001], relative to more consistent learning and recall scores across the four lists by the 2-Back condition [F(3,147) = 2.44; p = 0.07]. In other words, we did not observe an actual decrease in the mean number of items recalled by the 2-Back group across the four word lists, but an increase in items recalled by the 0-Back group. This is surprising, given that participants were presented with a different, novel, word list at each block. To explore this further, paired samples t-test revealed that participants in the 0-Back group recalled significantly more items from List C relative to List B (p<0.01), and from List D relative to List C (p<0.01). A comparable increase was not evident in the 2-Back conditions (List B to C: p = 0.35; List C to D: p = 0.07), although this latter contrast does reach marginal significance. No significant change was observed between List A and List B in either condition (0-Back: p = 0.25; 2-Back: p = 0 .68).

More specifically, these significant changes in the 0-Back group represents a change in free recall ability from an average of approximately seven (7/15) items per group at baseline (List A) to a mean of approximately nine (9/15) items from List D. A far more modest increase of approximately half an extra item (i.e. from approximately 7/15 items from List A to 7.5/15 items from List D) in the 2-Back group. The maintenance of discrete pieces of information within ones current mental set has a limited capacity which is in general accepted to average approximately 7 items [46]. The integration of these elements into a coherent, long-term memory representation does not succumb to such capacity restraints and could therefore easily support the encoding and recall of nine disparate items. We hypothesise that the enhanced mnemonic performance observed in the 0-Back condition, may reflect an incremental shift from a working memory based cognitive/neural strategy to a potentially more successful associative memory strategy; a shift which fails to occur when participants are required to specifically engage in the 2-Back working memory task immediately prior to the verbal learning task.

Finally, in order to investigate the temporal profile associated with this mnemonic impairment following the performance of the 2-Back WM task, we divided the data from the 2-Back group into trials that were presented immediately after the cessation of the 2-Back task (i.e. the first five words presented to each participant from lists B, C, and D), and the trials that were presented late in the learning phase task (i.e. the last five words presented to each participant from lists B, C, and D). Interestingly, no performance differences were observed (when compared using a paired samples t-test) between these two epochs on any of the word lists (List A: t(49) = 1.46; p = 0.15; List B: t(49) = −0.63; p = 0.53; List C: t(49) = 1.47; p = 0.15; List D: t(49) = 0.28; p = 0.78; see Figure S4), suggesting that the detrimental effect of the 2-Back WM task on subsequent memory encoding did not differ across epochs, and therefore appeared to be sustained throughout the post 2-Back task block.

#### Hippocampal-dependent/Direct Recognition

When tested directly, participants in the 2-Back group recognised significantly fewer ‘seen’ words than those who had performed the 0-Back control task [F(1, 48) = 5.61; p<0.05; see Figure 5: Panel B]. This difference was further emphasised when false positive responses were subtracted from the total number of words correctly recognised [F(1, 48) = 10.8; p<0.01]; with this analysis again indicating that those who performed the 0-Back task were significantly more accurate at recognising target items than those in the 2-Back group.

#### Hippocampal-independent/Indirect Recognition

Here we observed a strikingly different pattern of results. Whilst, we observed a main effect of stimulus type [F(1,48) = 144.11; p<0.001] (as participants identified significantly more ‘seen’ than ‘unseen’ words), no main effect of Group [F(1,48) = 0.33; p = 0.57], nor Group x Word Type interaction [F(1,48) = 0.63; p = 0.80] were observed here. We thus found no evidence to suggest that participants, who had performed the 2-Back task prior to the verbal learning trials, were impaired at indirectly recalling the previously studied words. This was confirmed by further one-way ANOVAs which revealed no significant between-group differences in either the ‘seen’ [F(1, 48) = 0.14; p = 0.71] or ‘unseen’ [F(1, 48) = 0.55, p = 0.46] categories (see Figure 5: Panel C).

Finally, performance accuracy on both the 0-Back [99.25% (±0.6%)] and the 2-Back [85.09% (±13.3%)] tasks was high.

### Experiment 2

**Figure 6:**
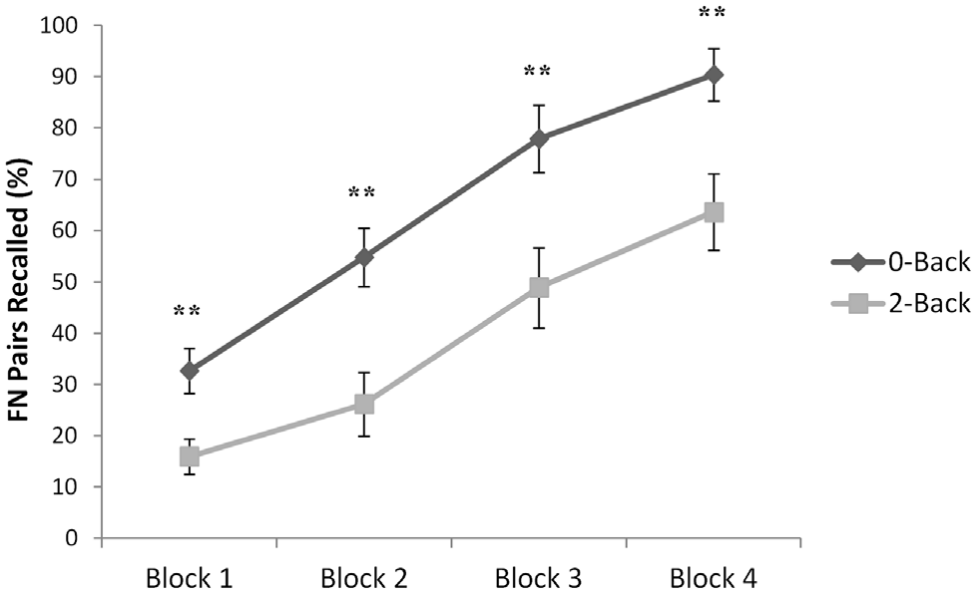
Results: Experiment 2. At each of the 4 epochs, participants who had previously performed the 2-Back task recalled significantly fewer face-name pairs than those who had previously performed the 0-Back control task. Error bars indicate SEM. ** *P*<0.01.

All participants exhibited a positive learning curve across the four learning and recall blocks, i.e. a main effect of trial [F(3,66) = 81.90; p<0.001]. Overall, learning was significantly better in the participants who performed the 0-Back, relative to those who performed the 2-Back task, prior to the face-name encoding phases [F(1,22) = 12.65; p<0.01]. No significant Group X Block interaction was observed [F(3,66) = 1.24; p = 0.30]. Additional one-way ANOVAs revealed that the 2-Back group recalled significantly fewer face-name pairs at each of the four learning and recall blocks: Block 1 [F(1,22) = 8.67; p<0.01], Block 2 [F(1,22) = 11.50; p<0.01], Block 3 [F(1,22) = 8.29; p<0.01], and Block 4 [F(1,22) = 9.02; p<0.01 (see Figure 6), than those in the 0-Back group. Again, the detrimental impact of the 2-Back task performance was sustained within each of the four face-name encoding blocks [i.e. the recall of the face-name pairs presented immediately after performance of the 2-Back task ceased (‘First Epoch’) did not differ significantly from the recall of the face-name pairs presented further away in time from the cessation of the 2-Back task (‘Last Epoch’): Block 1, t(10) = 0.61; p = 0.55; Block 2, t(10) = 1.55; p = 0.15; Block 3, t(10) = −0.76; p = 0.47, Block 4, t(10) = −0.89; p = 0.40; see Figure S5 for further details].

Finally, no performance differences were observed between the groups on the visual attention task [F(1,22) = 0.12; p = 0 .73] and performance accuracy on both versions of the *n*-Back task was high [0-Back = 99.4% (±1.0%); 2-Back = 89.2% (±9.8%)].

### Experiment 3

**Figure 7:**
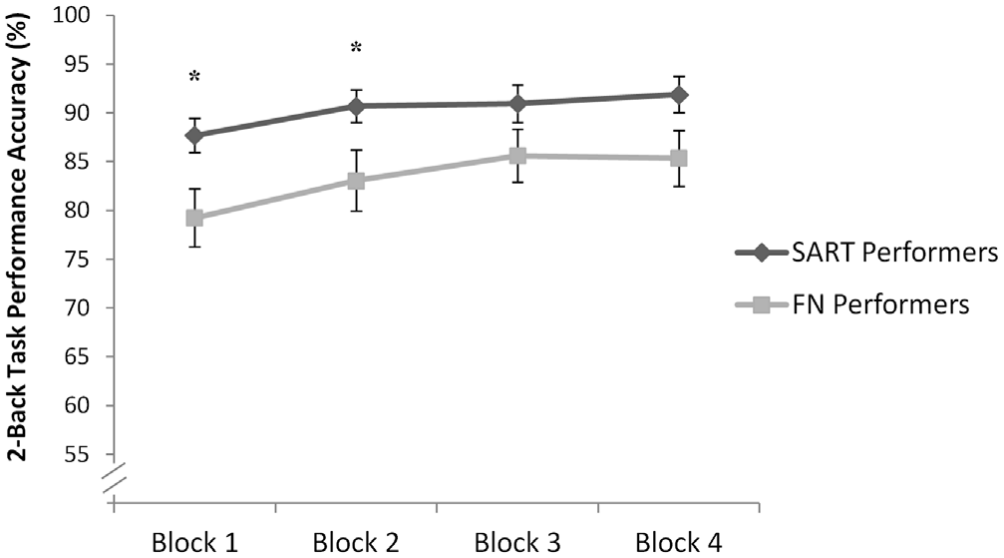
Results: Experiment 3. Overall, participants in the experimental group, who performed the Face-Name Learning and Recall task, performed significantly worse on the subsequent 2-Back Working Memory task than those in the control group, who performed the SART instead of the Face-Name task. This difference was significant at the first and second 2-Back Task blocks (but not at blocks 3 and 4). Error bars indicate SEM. * *P*<0.05.

Performance accuracy on the 2-Back task was compared between the participants who had performed the face-name learning and recall task and those who had performed the Sustained Attention to Response Task (SART). A significant main effect of both block [F(3,162) = 5.41; p<0.01] and group was observed [F(1,54) = 5.35; p<0.05] (see Figure 7). This between-group difference reflected significantly less accurate performance of the 2-Back task by participants in the experimental group (i.e. those who had first performed the face-name task) relative to the control group (i.e. those who had first performed the SART) at blocks 1 and 2 [Block 1: F(1,54) = 5.82; p<.05; Block 2: F(1,54) = 4.39; p<.05; Block 3: F(1,54) = 2.53; p = .12; Block 4: F(1,54) = 3.56; p = .06]. No group X block interaction was observed [F(3, 162) = 0.45; p = 0.72].

## Discussion

The results of Experiment 1 demonstrate that, as predicted, prior performance of the 2-Back working memory task impaired the recollection of to-be-remembered verbal material relative to participants who had performed the 0-Back control task. Moreover, this manipulation selectively impaired hippocampal-related memory, whilst sparing hippocampal-independent mnemonic processes [47]. Thus performance of the 2-Back working memory task altered hippocampal-dependent mnemonic processes in such a way that neurologically-intact young participants displayed an impaired memory profile reminiscent of that typically observed in patients with hippocampal lesions [34], [35], [48]. These initial observations were further supported by the results of Experiment 2, which again demonstrated that antecedent performance of the 2-Back tasks appeared to significantly attenuate the learning and recall of novel face-name pairs. Moreover, this effect was larger and more striking than that observed in Experiment 1. This difference is unsurprising given that the ability to form new associations is believed to be highly dependent of the integrity of these MTL regions (for review see [49]), and is disproportionally impaired following MTL damage ([50], but see also [51]). Finally, we observed a reduction of 2-Back WM task accuracy in participants who engaged in prior, sustained, face-name learning (a heavily hippocampal-dependent task) relative those who performed the SART (a task which does not appear to engage the hippocampus). Thus, in Experiments 1 and 2 we were able to selectively impair hippocampal-supported memory in healthy young adults using a simple behavioural manipulation (i.e. the prior performance of a task with an inhibitory mnemonic component), while the results of Experiment 3 mirrored these earlier findings, albeit in reverse order. As such, subsequent task performance in all three experiments was attenuated when the mnemonic goals of the preceding task competed with those of the subsequently performed task.

We propose that the attenuation of performance on the subsequent task in each experiment was a result of a failure to switch from the mnemonic and neural task-set primed during the performance of the initial task [which in all cases had a conflicting functional (memory) and anatomic (MTL) profile to that of the preceding task] to the more efficient and now appropriate task set. Similar theories have been proposed in the past to account for residual task-switch effects which persist even when participants are given ample time to prepare for the upcoming tasks. For example, Allport, Styles, and Hsieh's ‘task set inertia’ theory proposed that task-sets persist from one trial to the next, and that impaired performance on a post-switch trial is attributable to the activation of this now task-inappropriate task, and the persistent inhibition of the now task-appropriate task set, during performance of the pre-switch task [52]; see also [6], [53], [54], [55], [56]). What differed here was that we predicted and observed these task-switch deficits by systematically interweaving tasks which have conflicting neural profiles within the MTL (relative to tasks that do not), and therefore effectively constructed a suboptimal ‘neural task set’ in which our tasks of interest were performed.

Whilst cognitive fatigue associated with the performance of the 2-Back task is one potential explanation for the results of Experiments 1 and 2, we suggest that this is highly unlikely, given that the participants performed this task at a consistently high level across the task blocks (with approximately 90% accuracy). Moreover, each task block was short, lasting only two and a half minutes, and prior to starting Experiment 2, participants received sufficient training at the 2-Back task to ensure that they were not unduly taxed during the experimental 2-Back blocks. In addition, no performance differences between the 0-Back and 2-Back groups were observed in Experiment 2 on the visual attention task and generalised cognitive slowing due to fatigue should have been evident across all domains and not limited to learning and recall tasks. This hypothesis therefore struggles to account for the above selective pattern of deficits observed following the performance of the 2-Back WM task. Fatigue-related explanations that revolve around a loss of voluntary control have not however been explicitly tested and they present an intriguing framework for future studies [57], including paradoxical effects on aging [58] which might prove to be an interesting variable for studies of age-related memory decline.

Alternatively, it could be argued that participants in the 0-Back condition were unfairly advantaged by the lower cognitive demands associated with the performance of this task as they could potentially rehearse the to-be-recollected material during the task blocks, whereas the 2-Back participants had less opportunity to do this. We addressed this issue in Experiment 1, whereby participants viewed a different word list at each learning and recall interval. Thus any impairment observed on the subsequent and immediate recall of lists B, C or D could not be accounted for in this way, as participants had not yet been presented with the to-be-remembered material during the *n*-Back task blocks. We therefore propose that these results are best explained in terms of the hypothesised carry-over of an inappropriate mnemonic, and corresponding ‘neural task-set’, which is suboptimal for the performance of the task currently at hand (in this case a verbal learning and recall task). Interestingly, the observation that performing the 2-Back task appeared to prevent an equivalent enhancement of learning across the four word lists, observed in the 0-Back condition but absent in the 2-Back group, suggests that the 2-Back participants were unable to enhance their mnemonic performance in the same way that the 0-Back participants were. We speculate that this enhancement may be driven by the recruitment of more associative memory strategies; processes which are evidently and significantly disrupted via the prior performance of the 2-Back task (Experiment 2).

Given the self-paced nature of these experimental paradigms, it was not possible to systematically explore the temporal nature of this effect. We predict that the detrimental impact of prior 2-Back WM task performance on subsequent MTL memory processes (and vice versa) is sustained as long as one can sufficiently perform the task at hand (even if performance is being supported by a sub-optimal cognitive strategy or neural network). A switch to the now appropriate task-set must surely occur if participants are unable to perform the subsequent task using the carried over ‘neural task-set’. Alternatively, it is possible that there is a limited time period during which such effects are evident, which commences immediately after the completion of the initial task, persists for a finite duration, and is independent of whether performance of the switch task is partially supported by the previous, now inappropriate neural task-set, or not. Future studies which systematically manipulate the time period between the end of the preceding task and the start of the subsequent task are needed to fully investigate this possibility. However, given that we observed no obvious differential impact on subsequent memory for items (Experiment 1) or face-name pairs (Experiment 2) presented immediately after the cessation of the 2-Back WM task, relative to those presented furthest away in time from the cessation of the 2-Back task, suggests that such finite time-frame is unlikely to exist. In addition, the fact that learning continued to occur in both Experiments 1 and 2 following 2-Back task performance (although at a slower rate than after 0-Back task performance), supports the idea that learning is occurring through a sub-optimal (perhaps frontally-driven) memory network. The use of functional neuroimaging techniques could offer insight into the exact neural processes being engaged and disengaged during these intriguing cognitive manipulations, and would enable the exact relationship between the deactivation of the MTL during 2-Back WM task performance and subsequent hippocampal-mnemonic ability (and vice versa) to be more precisely elucidated.

The interpretation of task-dependent deactivations, or the negative BOLD responses observed in fMRI, remains a controversial issue. This is largely due to the multiple hypotheses that have been proposed to account for their presence [59]. One such hypothesis, the neuronal inhibition hypothesis, suggests that a significant proportion of these events represent the inhibition of neuronal firing patterns within the region in question [60]. This rationale is evident in numerous cognitive neuroimaging papers where task-specific deactivations observed in regions remote from the focal activation are considered to represent a suppression, inhibition, or ‘gating’ of the cognitive processes linked to the inhibited regions [61], [62]. This is believed to occur as the cognitive function associated with the deactivated cortex is either not required or competes with the demands of the particular task [20], [21], [62], [63] and such interpretations therefore offer a task-appropriate down-regulation or inhibitory explanation of these events.

Significantly, a number of neurophysiological studies have attempted to directly explore the expression of negative BOLD using physiological phenomenon linked to neuronal inhibition. One such study observed strong negative BOLD signals in ipsilateral primary sensorimotor cortex and adjacent subcortical regions during a unilateral finger tapping paradigm. Given that these regions were activated when the opposing hand performed the same movement, the authors concluded that this ipsilateral negative BOLD was linked to the transcallosal inhibition of the region responsible for the identical movement on the opposite side of the body ([64] see also [65]). Similarly, Kastrup and colleagues observed a decreased BOLD signal in ipsilateral primary somatosensory cortex during unilateral electric stimulation of the right median nerve [66]. Interestingly, the extent of this signal change in the area representing the index finger correlated with an increase in the current perception threshold associated with the contralateral and unstimulated index finger. This compelling psychophysiological evidence showed for the first time a measureable behavioural consequence of a functional inhibition expressed as a decrease in the BOLD signal within the somatosensory system. More direct evidence for the neuronal inhibition hypothesis was established using simultaneous fMRI and electrophysiological recordings. Here it was demonstrated that the negative BOLD response expressed in monkey visual area V1 was associated with decreases in neuronal activity relative to spontaneous activity and correlated with decreases in local field potentials, multiunit and spiking activity [59]. Based on this data the authors estimated that neuronal inhibition accounted for more than 60% of all observed negative BOLD.

The inhibitory mnemonic component inherent to this version of the 2-Back task utilised in the above paradigms is, at least at a cognitive level, consistent with the neuronal inhibition account of such deactivations [19] and the above data fit best with such an interpretation. More specifically, our data speculatively indicates that the putative deactivation of the MTL associated with 2-Back WM task performance is based on a physiologically-real inhibitory neural phenomenon which, as previously demonstrated within the somatosensory system [66], appears to have observable functional consequences on behaviour. The above results also suggest that these behavioural consequences are temporally-sustained and can exert their influence on future task performance (e.g. on subsequent mnemonic performance; see also [67]). This, perhaps surprising effect, appears to be robust and consistent across a number of cognitive paradigms (i.e. across both verbal items and paired-associate learning). As such, it is conceivable that the systematic manipulation of regional deactivations (using tasks such as the 2-Back WM task) may represent an important, temporally- and spatially-constrained methodology that could enable real-time interventions in the execution of fundamental cognitive processes. This possibility is supported by the observation that these MTL deactivations are highly consistent in healthy young adults during the performance of the 2-Back WM task [in fact, they are so reliable that their absence can be used as a marker of suboptimal task performance in patients suffering from, or at risk of developing, schizophrenia [19], [21], [24], [25]]. This could therefore represent a significant and new methodological approach, where the task-induced systematic deactivation of specific brain regions could be used to generate “virtual” and reversible brain lesions in the neurologically-intact brain, and would present an opportunity to investigate cognitive processes in the absence of the engagement of particular structures. This has obvious implications for the furthering of current theoretical models of human neuro-cognitive processing systems.

Regardless, however, of the neural profile of the tasks in question, or of the appropriate interpretations of these neuroimaging signals, we propose that the above findings strongly support the idea that memory is not a static ability. We argue that while alternate memory tasks may share cognitive processes, many memory tasks recruit conflicting cognitive sub-systems and supporting neural networks which can impact on one another; rendering an individual's memory ability a product, not only of an individuals' inherent abilities, but also of the current neural context (or ‘neural task-set’) in which the task of interest is being performed. In this way, we show that memory can effectively be down-regulated via the temporal environment in which it is functioning. Thus, memory should not be viewed as a static, inherent property, but as a multifaceted, dynamic and mutable process.

## Supporting Information

Figure S1 **Verbal Recollection Task.** *Panel A*: *Explicit Verbal Learning and Recall*. During the encoding phase, participants viewed each word on screen for approximately 1000 ms followed by an ISI of 1000 ms. Participants then verbally engaged in free recall. *Panel B*: *Hippocampal-Dependent Recognition*. During this phase, participants were required to identify the words that they had encountered during the learning and recall trials (i.e. ‘seen’ words) and those that they had not (i.e. ‘unseen’ words). Stimuli were presented on-screen until a response was made and this was followed by a fixation cross (500 ms). *Panel C*: *Hippocampal-Independent Recognition*. Participants attempted to read each of the 120 masked stimuli aloud. Each stimulus remained on screen for a total of 1000 ms, followed by a fixation cross (500 ms). As with the explicit recognition paradigm, 60 of the 120 stimuli had been previously studied during the learning trials (i.e. ‘seen’ trials) and 60 were novel (i.e. ‘unseen’ trials).
(TIF)

Figure S2 **Face-Name Task.** *Panel A: Face-Name Encoding*. Participants viewed each face-name pair for 3500 ms (ISI = 500 ms), during which time they attempted to memorise the name corresponding to each face. *Panel B: Visual Attention Task*. Participants focused on the fixation cross and pressed a button, as quickly as possible, every time it changed to a solid black circle. *Panel C: Face-Name Retrieval*. Participants were presented with each of the eight previously viewed faces (in random order). They were required to vocally recall the name corresponding to each face. Each face was viewed for 3500 ms (ISI = 500 ms).
(TIF)

Figure S3 **Sustained Attention to Response Task (SART).** In this task participants are presented a random series of digits; ‘1’ through to ‘9’. Each digit remained on screen for a duration of 250 ms (ISI = 900 ms) during which time participants were required to make a button-press response for each presented digit (‘go trial’), except when presented with the digit ‘3’ (‘no-go trial’).
(TIF)

Figure S4 **Experiment 1: Verbal Recall – Epoch Analysis.** The verbal recall data from the 2-Back group was divided into epochs, whereby the first epoch contained the trials that were presented immediately after the cessation of the 2-Back task (i.e. the first five words from each word list presented to each participant), and the last epoch contained the trials that were presented last in the encoding phase (i.e. the last five words from each word list presented to each participant). Subsequent recall of the words contained in the first and last epochs was compared using paired samples t-tests. No performance differences were observed (List A: p = 0.15; List B: p = 0.53; List C: p = 0.15; List D: p = 0.78).
(TIF)

Figure S5 **Experiment 2: Face-Name Recall – Epoch Analysis.** The face-name data from the 2-Back group was divided into two epochs, whereby the first epoch contained the subsequent memory scores of face-name pairs presented immediately after the conclusion of the 2-Back condition (i.e. the first four face-name pairs presented in each block), and the last epoch contained the subsequent memory scores of face-name pairs presented in the later block (i.e. the last four face-name pairs presented in each block). When mnemonic performance on the first and last epochs was compared (using paired-samples t-tests), no significant differences were observed between the number of face-name pairs subsequently recalled from the first relative to the last epoch, across any of the individual task blocks (Block 1: p = 0.55; Block 2: p = 0.15; Block 3: p = 0.47; Block 4: p = 0.40).
(TIF)
