# Supplementary figures and images for: Suppressing the Encoding of New Information in Memory: A Behavioral Study Derived from Principles of Hippocampal Function
Source: PLoS One. 2013 Jan 16;8(1):e50814. doi: 10.1371/journal.pone.0050814 (PMC3547015; doi:10.1371/journal.pone.0050814)

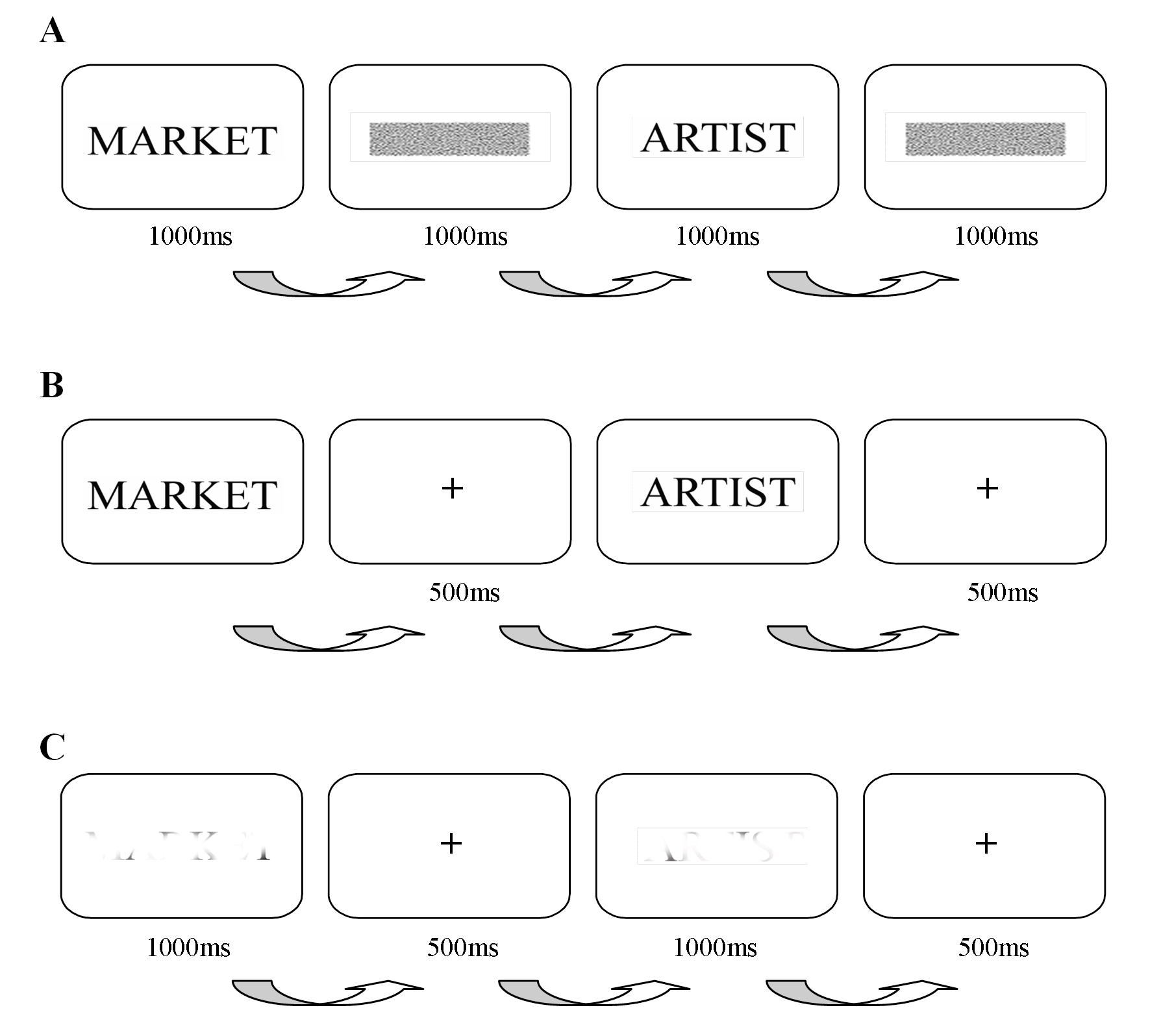

Supplement: Figure S1 — Verbal Recollection Task. Panel A: Explicit Verbal Learning and Recall. During the encoding phase, participants viewed each word on screen for approximately 1000 ms followed by an ISI of 1000 ms. Participants then verbally engaged in free recall. Panel B: Hippocampal-Dependent Recognition. During this phase, participants were required to identify the words that they had encountered during the learning and recall trials (i.e. ‘seen’ words) and those that they had not (i.e. ‘unseen’ words). Stimuli were presented on-screen until a response was made and this was followed by a fixation cross (500 ms). Panel C: Hippocampal-Independent Recognition. Participants attempted to read each of the 120 masked stimuli aloud. Each stimulus remained on screen for a total of 1000 ms, followed by a fixation cross (500 ms). As with the explicit recognition paradigm, 60 of the 120 stimuli had been previously studied during the learning trials (i.e. ‘seen’ trials) and 60 were novel (i.e. ‘unseen’ trials). (TIF) [file pone.0050814.s001.tif]

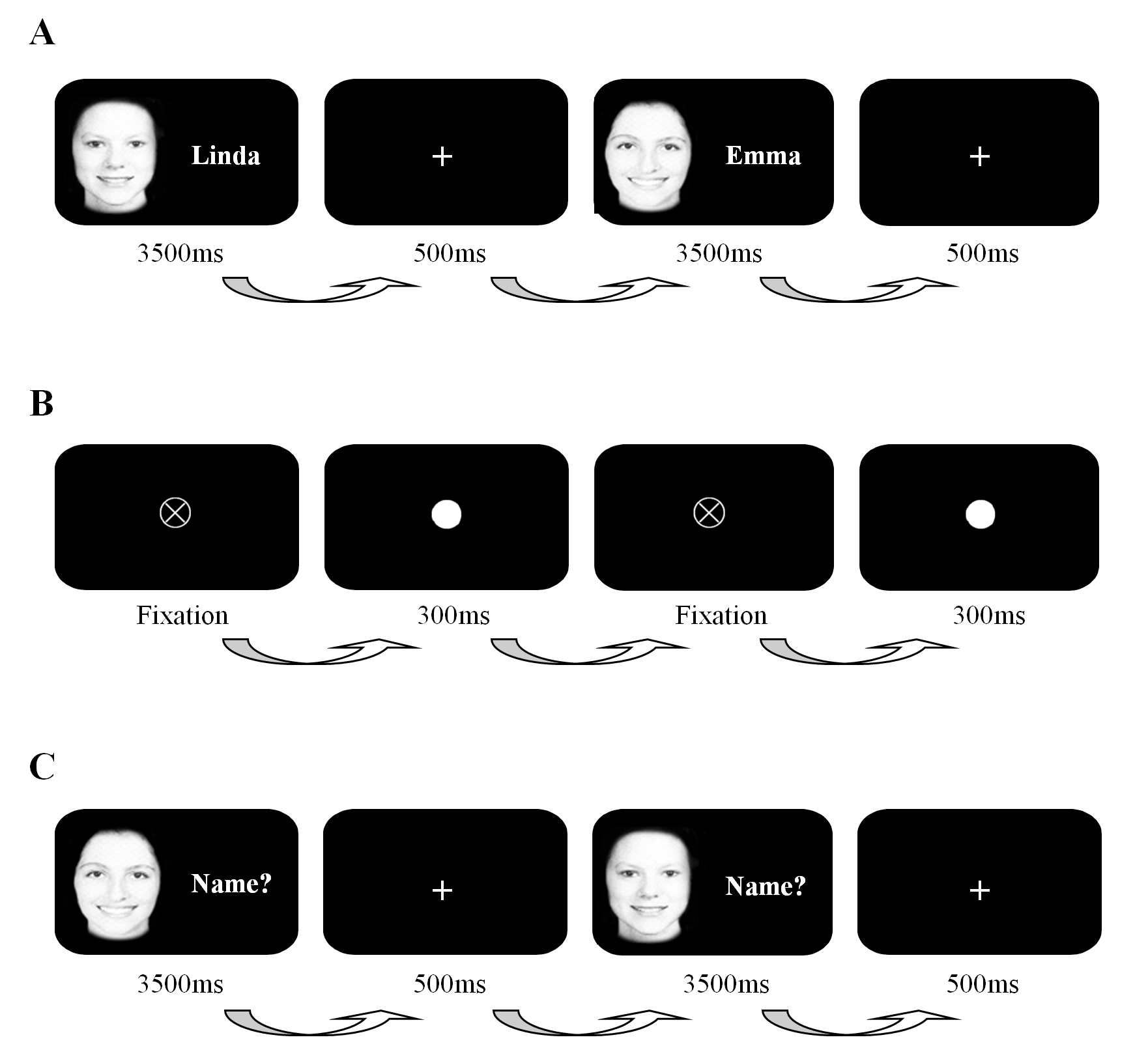

Supplement: Figure S2 — Face-Name Task. Panel A: Face-Name Encoding. Participants viewed each face-name pair for 3500 ms (ISI = 500 ms), during which time they attempted to memorise the name corresponding to each face. Panel B: Visual Attention Task. Participants focused on the fixation cross and pressed a button, as quickly as possible, every time it changed to a solid black circle. Panel C: Face-Name Retrieval. Participants were presented with each of the eight previously viewed faces (in random order). They were required to vocally recall the name corresponding to each face. Each face was viewed for 3500 ms (ISI = 500 ms). (TIF) [file pone.0050814.s002.tif]

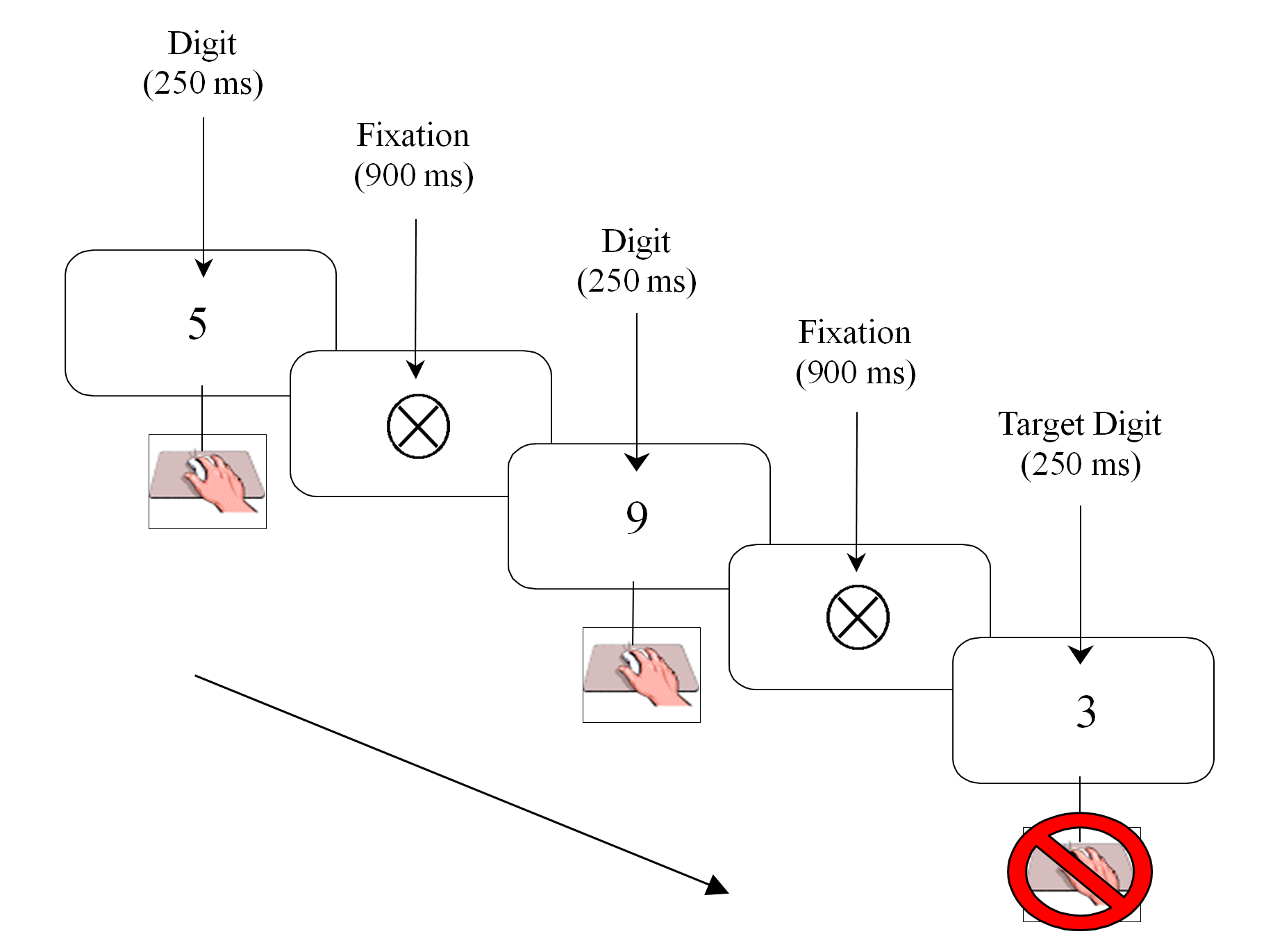

Supplement: Figure S3 — Sustained Attention to Response Task (SART). In this task participants are presented a random series of digits; ‘1’ through to ‘9’. Each digit remained on screen for a duration of 250 ms (ISI = 900 ms) during which time participants were required to make a button-press response for each presented digit (‘go trial’), except when presented with the digit ‘3’ (‘no-go trial’). (TIF) [file pone.0050814.s003.tif]
